# Establishment of a Transgenic Zebrafish Line for Superficial Skin Ablation and Functional Validation of Apoptosis Modulators In Vivo

**DOI:** 10.1371/journal.pone.0020654

**Published:** 2011-05-31

**Authors:** Chi-Fang Chen, Che-Yu Chu, Te-Hao Chen, Shyh-Jye Lee, Chia-Ning Shen, Chung-Der Hsiao

**Affiliations:** 1 Institute of Bioscience and Biotechnology, National Taiwan Ocean University, Keelung, Taiwan; 2 Department of Bioscience Technology, Chung Yuan Christian University, Chung-Li, Taiwan; 3 National Museum of Marine Biology and Aquarium, Pingtung, Taiwan; 4 Institute of Zoology, National Taiwan University, Taipei, Taiwan; 5 Genome Research Center, Academia Sinica, NanKang, Taipei, Taiwan; 6 Center for Nanotechnology, Chung Yuan Christian University, Chung-Li, Taiwan; University of Birmingham, United Kingdom

## Abstract

**Background:**

Zebrafish skin is composed of enveloping and basal layers which form a first-line defense system against pathogens. Zebrafish epidermis contains ionocytes and mucous cells that aid secretion of acid/ions or mucous through skin. Previous studies demonstrated that fish skin is extremely sensitive to external stimuli. However, little is known about the molecular mechanisms that modulate skin cell apoptosis in zebrafish.

**Methodology/Principal Findings:**

This study aimed to create a platform to conduct conditional skin ablation and determine if it is possible to attenuate apoptotic stimuli by overexpressing potential apoptosis modulating genes in the skin of live animals. A transgenic zebrafish line of Tg(*krt4:NTR-hKikGR*)^cy17^ (killer line), which can conditionally trigger apoptosis in superficial skin cells, was first established. When the killer line was incubated with the prodrug metrodinazole, the superficial skin displayed extensive apoptosis as judged by detection of massive TUNEL- and active caspase 3-positive signals. Great reductions in NTR-hKikGR^+^ fluorescent signals accompanied epidermal cell apoptosis. This indicated that NTR-hKikGR^+^ signal fluorescence can be utilized to evaluate apoptotic events in vivo. After removal of metrodinazole, the skin integrity progressively recovered and NTR-hKikGR^+^ fluorescent signals gradually restored. In contrast, either crossing the killer line with testing lines or transiently injecting the killer line with testing vectors that expressed human constitutive active Akt1, mouse constitutive active Stat3, or HPV16 E6 element displayed apoptosis-resistant phenotypes to cytotoxic metrodinazole as judged by the loss of reduction in NTR-hKikGR^+^ fluorescent signaling.

**Conclusion/Significance:**

The killer/testing line binary system established in the current study demonstrates a nitroreductase/metrodinazole system that can be utilized to conditionally perform skin ablation in a real-time manner, and provides a valuable tool to visualize and quantify the anti-apoptotic potential of interesting target genes in vivo. The current work identifies a potential use for transgenic zebrafish as a high-throughput platform to validate potential apoptosis modulators in vivo.

## Introduction

The epidermis is the outermost and largest structure covering the body. It plays a role in protection against mechanical injury forming a major barrier to deleterious environmental agents. The mammalian epidermis comprises four major layers: the basal layer, spinous layer, granular layer and cornified layer. The stem cells predominantly distribute in the basal layer and mitotic division of these cells generates other differentiated cells. In contrast to mammals, fish epidermis is relatively thin and simple. During early development, the fish epidermis has only two layers; the outermost enveloping layer (EVL) and inner epidermal basal layer (EBL), which provide a first-line defense system against pathogens. Fish epidermis contains ionocytes and mucous cells that aid secretion of acid/ions or mucous through the skin [1]. At adult stage, fish skins differ in thickness and cell type among species [2].

When skin is exposed to environmental stress this triggers an apoptotic response to prevent accumulation of oncogenic mutations in its layers. Activation of repairing pathways ensues in order to recover skin integrity [3]. In recent decades, scientists identified involvement of genes in modulation of skin cell apoptosis using *in vitro* culture methods. However, some drawbacks such as dissimilarity to real skin physiology [4] make the approach less informative. The recent solution is to validate the function of potential apoptosis modulators in skin by generating a genetically modified mouse, utilizing transgenic (gain-of-function) or knock-out (loss-of-function) approaches, and then subsequently challenging them with external stress to evaluate the skin response. These combinational strategies led to successful identification of several genes including Bcl-xL [5], Nrf2 [6], hsp70 [7], Stat3 [8], Akt1 [9], survivin [10], [11], galectin-3 [12], RhoB [13] and peroxiredoxin 6 [14] that suppress skin apoptosis upon receiving UVB- or chemical-induced damage. The clinical challenge is to discover new promoters of skin survival against environmental stresses and to develop a new generation of skin anti-aging or anti-apoptotic therapeutic reagents [15]. Development of a high-throughput platform to screen for apoptosis modulators in living animals would, therefore, significantly accelerate progress in validating gene function *in vivo*.

The zebrafish is a powerful model organism that has been extensively used to study developmental mechanisms, genetics, apoptosis, and human disease in recent years [16], [17], [18]. The advantage of utilizing zebrafish to study apoptosis modulators in skin is that fish skin does not have a true stratified epithelium and lacks a stratum corneum. The outermost skin layer in fish consists of living cells rather than a dead cornified layer as in mammals. Therefore, the simple and fragile architecture of fish skin provides ideal tissue to evaluate the impact of environmental stress derived from mechanical injury [19], [20], prey biting [21], pathogen infection [22], ultraviolet (UV) irradiation [23], [24], algae bloom [25], [26] or long-term exposure to eco-toxins [27]. A recent study by Pai and Chen [28] used a transgenic approach to identify that overexpression of prothymosin alpha in zebrafish skin attenuated cell death caused by UV irradiation. This strategy demonstrated that evaluation of apoptosis modulator function after receiving damage signals *in vivo* is possible by adapting a similar approach to that used in the mouse skin system.

This study aimed to a create a specific platform to conduct skin ablation by generating a transgenic zebrafish with a skin-specific promoter to drive nitroreductase (NTR), and then adding metrodinazole (Met) to cause specific ablation of superficial skin cells. NTR is a non-toxic reductase isolated from *Escherichia coli* converted into toxic form when exposed to Met. Metabolic Met behaves as a DNA interstrand cross-linking agent to exert a cytotoxic effect and induce apoptosis. Recently, the wild-type NTR protein has been engineered into a more powerful version, by codon-optimization [29] or amino acid mutation [30], [31], which greatly enhanced its cytotoxicity when exposed to CB1954 or Met substrates. Therefore, the NTR/Met cell ablation technique has been successfully applied in many vertebrate species [32], [33], [34], [35] and displays superior and more reliable results compared to other cell ablation systems [36], [37], [38], [39]. Research groups successfully used the NTR/Met cell ablation system to stimulate ablation of embryonic pancreatic beta cells [40] and induce gonadal dysgenesis in zebrafish [41], [42]. In this study, a transgenic line of Tg(*krt4:NTR-hKikGR*)^cy17^ (killer line) was established which overexpressed the NTR-hKikGR fusion protein under the control of the skin-specific *krt4* promoter. Epidermal apoptosis could be induced by exposing the killer line embryos or adults to Met. Apoptotic cell death specifically occurred in the NTR-hKikGR-expressing cells, accompanied by the loss of fluorescent appearance. The zebrafish binary system, which combines killer and testing lines, therefore provides a unique quantitative and fast tool to study the signaling molecules which modulate skin apoptosis in living animals.

## Results

### Characterization of Zebrafish Skin Cell Organization and a Skin-Specific Promoter

**Figure 1:**
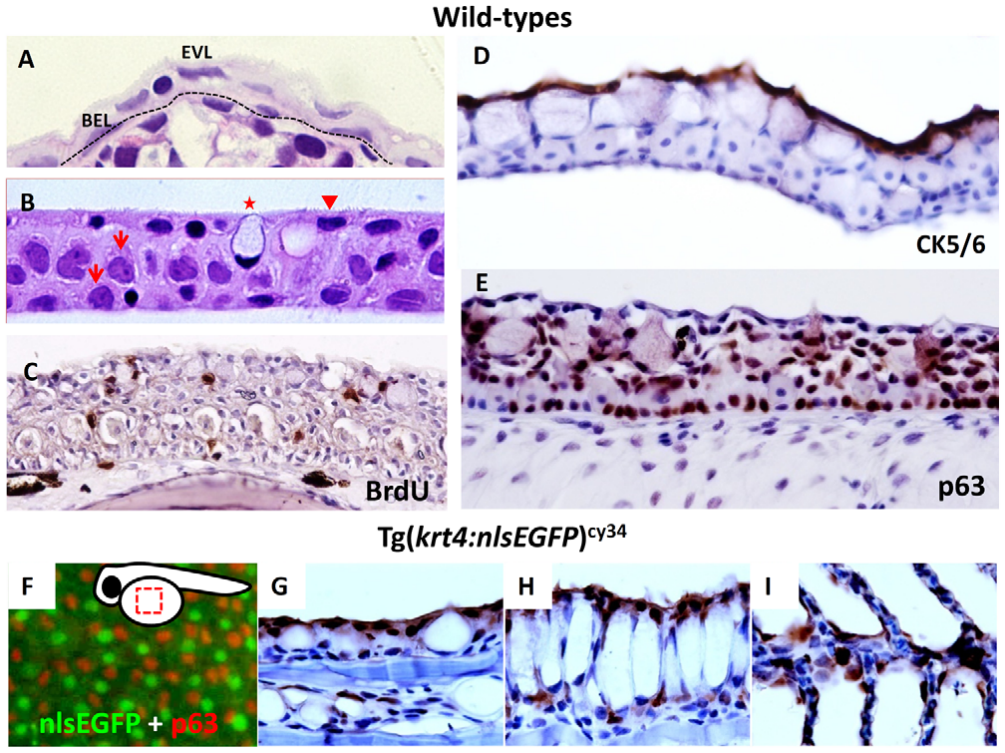
The *krt4* promoter can target transgene expression in the superficial skin layer in zebrafish. (A) Plastic section at 2 µm thickness showing that zebrafish skin, at 5 days post-fertilization, consists of an outer enveloping layer (EVL) and inner basal epidermal layer (BEL). The basement membrane is highlighted by the dotted line. (B) Plastic section at 2 µm thickness showing that adult zebrafish (aged at 3 months) skin consists of three major layers, including the superficial cells (arrowhead), middle and basal cells (arrows), and mucous cells (asterisk). (C) BrdU incorporation experiment showing that most skin layers in adult zebrafish are mitotically active. The BrdU^+^ cells (brown signals) can be detected in most skin layers at adult stage. (D) CK5/6 and (E) p63 antibodies differentially label the superficial skin layer and the putative epidermal stem cells in adult zebrafish skin, respectively. For immunohistochemistry, the 5 µm thick paraffin sections were immunostained with antigen-specific antibodies and visualized with DAB coloring substrate (brown). To visualize the cell morphology, the slides were counterstained with hematoxylin (blue). (F) Whole-mount immunostaining of p63 (red) on Tg(*krt4:nlsEGFP*)^cy34^ (green) embryonic yolk aged at 24 hpf, showing that the *krt4* promoter targets the outermost EVL. The relative position of the captured image is highlighted at the upper right corner. (G–I) Immunohistochemistry of GFP (brown) on paraffin sections derived from Tg(*krt4:nlsEGFP*)^cy34^ aged at 3 months. The positive signals (brown) show that the *krt4* promoter targets the superficial layer in the skin (G), esophagus (H) and gill (I) at adult stage.

Prior to performing skin ablation, the basic architecture of zebrafish skin was characterized. Consistent with previous studies [43], [44], zebrafish larvae skin consisted of EVL and BEL (Fig. 1A). The sagittal section of fully adult zebrafish skin (age greater than 3 months) was also analyzed. Results showed that the thickness of the skin varies in different positions. The epithelium surrounding the head and jaw was thicker than in other regions (data not shown). In contrast, the epithelium covering the scales was much thinner and usually organized into three cell layers. At higher magnification, cell nuclei of the upper superficial layer were flat and elongated (Fig. 1B, arrow head) while cell nuclei of the middle and basal skin layer appeared round and much larger than those in the superficial layer (Fig. 1B, arrows). High glycoprotein content and positive Periodic Acid Schiff (PAS) staining (data not shown) characterized larger cells, oval in shape and with peripheral cell nuclei, as mucous cells (Fig. 1B, asterisk). Cell morphology suggested the upper skin layer might be the differentiated layer while the middle and basal layers might form the undifferentiated layer. To test this hypothesis, BrdU incorporation experiment was performed to investigate cell proliferation activity in different skin layers of adult zebrafish. Result showed that BrdU^+^ skin cells widely distributed in the middle and basal layers, while the upper superficial layer displayed very few BrdU^+^ signals in most cases (Fig. 1C). This result is consistent with the findings obtained from tritiated thymidine injection [2], showing that the middle and basal skin layers have strong cell division/proliferation potential in adult zebrafish. To further clarify the skin differentiation pattern at the molecular level, immunohistochemistry was performed on adult skin to label the putative differentiated skin cells (using CK5/6 antibody) and epidermal stem cells (using p63 antibody). CK5/6 antibody staining strongly labeled the keratinocytes of the superficial skin layer (Fig. 1D) while p63 antibody staining strongly labeled most of the cell nuclei in the basal and middle skin layers (Fig. 1E). The BrdU, CK5/6 and p63 immunoreactive patterns suggested that the superficial skin layer is the differentiated layer while the middle and basal layers are the undifferentiated stem cell layers in zebrafish.

To establish an *in vivo* animal model to investigate mechanisms regulating apoptosis of the superficial skin cells, a suitable promoter to drive the suicide/killer gene, specifically expressed in the superficial skin layer in a temporally and spatially controllable manner, had to be identified. Previous studies isolated several useful skin-specific promoters in zebrafish, such as *krt4* [45], *krt5* [46] and *krt18* [47]. However, the specificity of those promoters in skin has yet to be fully characterized. To test the specificity of the *krt4* promoter in zebrafish skin Tg(*krt4:nlsEGFP*)^cy34^ was initially created. It was confirmed that the zebrafish *krt4* promoter could specifically drive nucleus-targeted EGFP (Fig. 1F, green) express in the superficial EVL, but not the underlying p63^+^ basal cells (Fig. 1F, red), in the embryonic yolk area at 24 hours post fertilization (hpf). Results from immunohistochemistry with GFP antibody on paraffin sections of adult Tg(*krt4:nlsEGFP*)^cy34^ demonstrated that the *krt4* promoter can specifically target the superficial skin layer covering the scales (Fig. 1G), esophagus (Fig. 1H) and gill (Fig. 1I). This result indicated that the endogenous 2.2 kb *krt4* promoter could be useful to promote superficial skin ablation in the present study's experiment.

### Generation of Killer Line to Express NTR Suicide Gene in the Superficial Skin Layer

**Figure 2:**
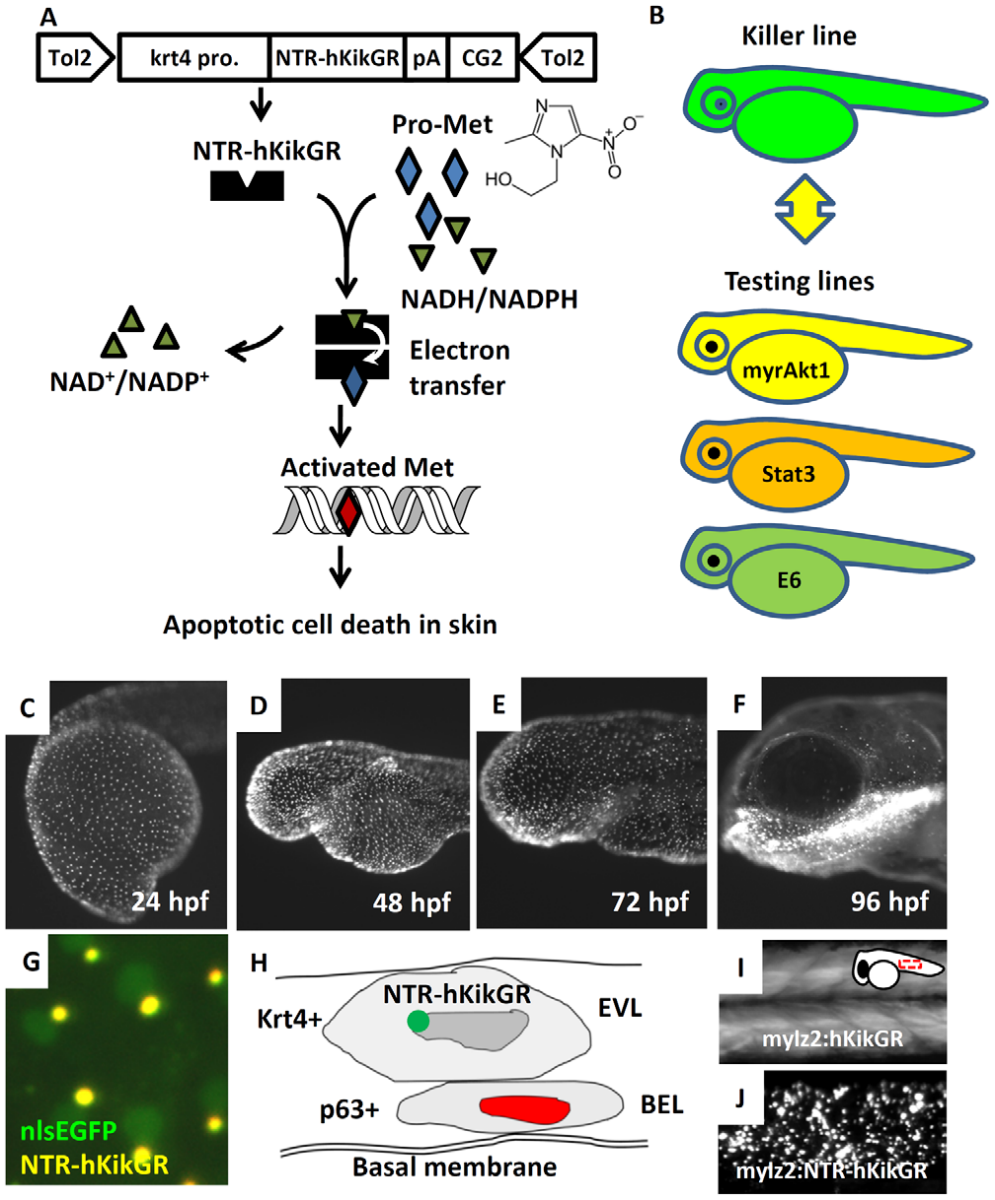
Establishment of Tg(*krt4:NTR-hKikGR*)^cy17^ killer line. (A) The work flow to conditionally ablate zebrafish skin using NTR/Met-mediated system. The superficial skin-specific *krt4* promoter controls NTR-hKikGR fusion protein. Tol2 transposon elements flank the whole transgene cassette and enhance the germ-line transmission rate. Dimerization of NTR-hKikGR transfers electrons from NADH/NADPH to Met prodrug. Activated Met crosslinks DNA and specifically triggers apoptotic death in skin. (B) The killer line carrying the krt4:NTR-hKikGR transgene was crossed with several testing lines which overexpress human constitutively active myrAkt1 (myrAkt1), mouse constitutively active Stat3 (Stat3), or HPV16 E6 (E6) genes. The double transgenics were then subjected to Met incubation to assay the potential function of apoptosis modulators. (C–F) The ontogenic expression pattern of NTR-hKikGR fusion protein in killer line aged from 24 to 96 hpf. (G) The living fluorescent signals detected in double transgenics from the crossing of the killer line and Tg(*krt4:nlsEGFP*)^cy34^ aged at 72 hpf show that the NTR-hKikGR^+^ signals (yellow) aggregated adjacently to the nlsEGFP^+^ (green) skin nucleus. (H) Model to illustrate the spatial distribution of NTR-hKikGR fusion protein in zebrafish embryo skin. (I) The native hKikGR protein displays cytoplasmic distribution pattern in the skeletal muscle of Tg(*mylz2:hKikGR*). The relative position of the captured image is highlighted at the upper right corner. (J) The NTR-hKikGR fusion protein aggregated in skeletal muscle of Tg(*mylz2:NTR-hKikGR*). EVL, enveloping layer; BEL, basal epidermal layer; Met, metrodinazole.

To enhance conditional targeted skin ablation in zebrafish, the mutated version of NTR (T41Q/N71S/F124T), the most sensitive version of NTR tested *in vitro*, was utilized [30]. In order to facilitate the transgenic line screening and monitor the dynamic expression of NTR in real-time, in-frame fused NTR with hKikGR photoconvertiable fluorescent protein at the C-terminus (hereafter, recognized as NTR-hKikGR) was used. The design of the transgenic cassette, mechanism of NTR/Met ablation system, and strategy for screening of apoptosis modulators using the killer/testing line binary system are illustrated in Figs. 2A and 2B. 15 independent lines out of 92 putative founders carrying the krt4:NTR-hKikGR transgene were identified (germ-line transmission rate = 16%). Among all transgenic lines identified, line number 17 (Tg(*krt4:NTR-hKikGR*)^cy17^, killer line) displayed the strongest fluorescent signal and exhibited normal skin development, with no evidence of cell toxicity in the absence of prodrug treatment. It was, therefore, selected to generate the homozygotic line for further skin ablation studies. In the killer line embryos, detection of the NTR-hKikGR^+^ signals first occurred at the 5-somite stage (data not shown). NTR-hKikGR^+^ signals in the skin gradually up-regulated and finally displayed robust expression in the pharyngeal arch epithelium (Figs. 2C–2F). The fluorescent signal of NTR-hKikGR fusion protein did not evenly distribute in the cytoplasm but aggregated into distinct small spots. These fluorescent dots seemed to localize in specific compartments of skin cells. To clarify the subcellular identity of NTR-hKikGR^+^ signals, the killer line was crossed with Tg(*krt4:nlsEGFP*)^cy34^, whose skin nucleus was highlighted with nlsEGFP fluorescent signals. Results showed that the NTR-hKikGR^+^ signals were located adjacent to the nuclei of EVL in the compound transgenics (Fig. 2G and also summarized in Fig. 2H). This unique localization of NTR-hKikGR fusion protein is not specific to the skin compartment, since NTR-hKikGR fusion protein demonstrated a similar dotted distribution pattern when driven by a muscle-specific *mlyz2* promoter (Fig. 2J).

### Loss of NTR-hKikGR^+^ Signals in Met-Treated Killer Line as a Living Marker of Skin Apoptosis

**Figure 3:**
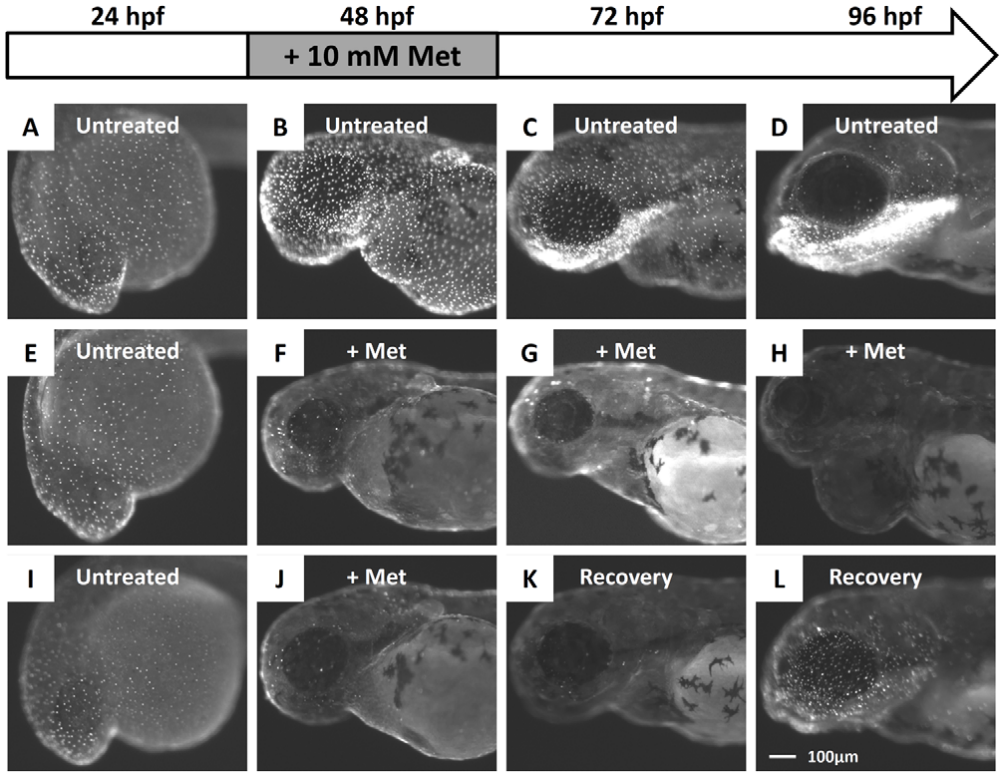
Administration of Met caused the killer line to lose the NTR-hKikGR+ fluorescent signals. (A–D) The ontogenic expression of NTR-hKikGR fusion protein in killer line embryos aged from 24 hpf to 96 hpf. (E–H) Consecutive incubation of killer line embryos with 10 mM Met, from 24 hpf to 96 hpf, caused the NTR-hKikGR^+^ fluorescent signals to gradually diminish by 48 hpf, totally disappear by 72 hpf, and show pericardial edema in Met-treated embryos by 96 hpf. (I–L) If Met was withdrawn and replaced with fresh fish water from 48 hpf onwards, the NTR-hKikGR^+^ fluorescent signals partially restored by 96 hpf. Scale bar = 100 µm in L (applies to A–L). The experimental design and work flow are illustrated at the top panel. Met, metrodinazole.

To validate whether the NTR/Met-based cell ablation system works properly in the killer line, killer line embryos were incubated with 10 mM Met from 24 to 48 hpf to ablate skin cells. Treatment was stopped by washing out Met after 48 hpf (Fig. 3, protocol indicated at upper panel). The NTR-hKikGR^+^ signals were monitored and photographed every 24 hours until 96 hpf. In the absence of Met (untreated), NTR-hKikGR^+^ fluorescent signals were robust throughout the entire experiment (Figs. 3A–3D). In the presence of Met, the NTR-hKikGR^+^ signals greatly diminished, and pericardial edema appeared at 96 hpf (Figs. 3E–3H). If Met was withdrawn after 48 hpf, the diminished NTR-hKikGR^+^ signals partially restored at 96 hpf (Figs. 3I–3L).

**Figure 4:**
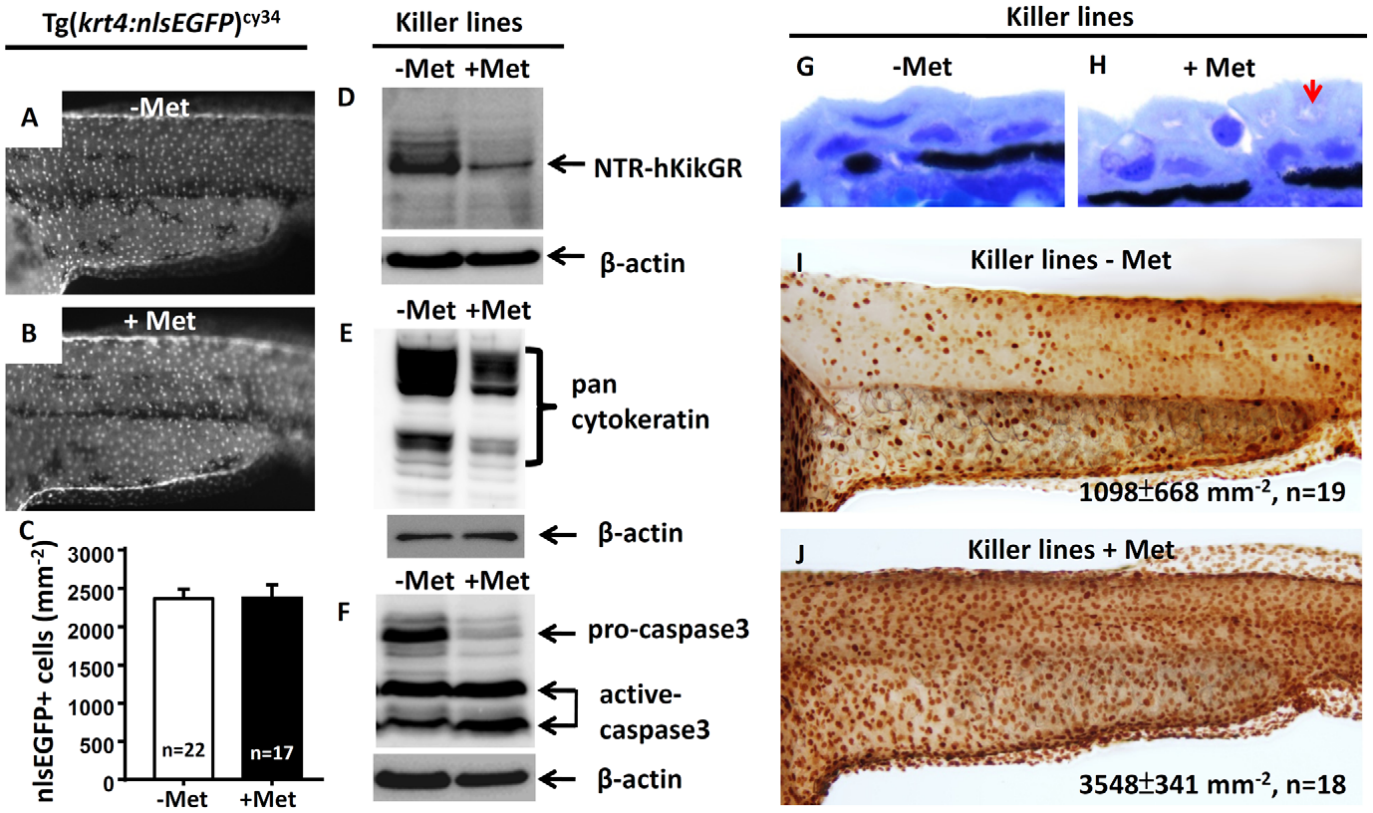
Apoptotic cell death mediates the loss of NTR-hKikGR^**+**^ fluorescent signals in Met-treated killer line. *krt4* promoter suppression does not mediate loss of NTR-hKikGR^+^ fluorescence in Met-treated killer line because downregulation of nlsEGFP^+^ fluorescence signal intensity (A and B) or nlsEGFP^+^ cells (C) in Tg(*krt4:nlsEGFP*)^cy34^ did not occur with or without Met treatment. (D–F) Western blot analysis showed that apoptotic cell death mediates the loss of NTR-hKikGR^+^ fluorescent signals in Met-treated killer line embryos because the relative expression levels of NTR-hKikGR fusion protein (D), pan-cytokeratin (E) and pro-caspase 3 (F) greatly reduced in Met-treated embryos. Note that the relative intensities of the cleavaged caspase 3-immunoreactive signals greatly increased in the Met-treated killer line (F). (G–H) Plastic sections at 2 µm thickness show the greatly compromised superficial skin integrity in Met-treated killer line embryos (arrow). Whole-mount TUNEL assay demonstrated significantly enhanced cell death signals in Met treated killer line embryos (J) compared to the untreated group (I). The cell number is presented as the mean±S.D. Student's *t*-test was used to make statistical comparisons between untreated (−Met) and Met-treated (+Met) killer lines. Met, metrodinazole.

This study hypothesized the following reasons for the loss of NTR-hKikGR^+^ signals: (1) activated Met suppressed the *krt4* promoter and blocked the expression of NTR-hKikGR^+^ fusion protein; (2) activated Met caused redistribution of NTR-hKikGR^+^ fusion protein back to the cytoplasm compartment causing it to lose its aggregated, high fluorescent nature; (3) activated Met triggered cell apoptosis in skin. The lack of significant difference in nlsEGFP^+^ cells between untreated (2365±124 mm^−2^, n = 22) and Met-treated Tg(*krt4:nlsEGFP*)^cy34^ (2372±177 mm^−2^, n = 17) ruled out the hypothesis that the same *krt4* promoter also drives the nlsEGFP reporter gene (Figs. 4A–4C). Western blot analysis showed that the relative amount of NTR-hKikGR fusion protein greatly reduced after Met treatment, indicating that the redistribution of NTR-hKikGR^+^ proteins was also not feasible (Fig. 4D). The loss of pan-cytokeratin provided further evidence of the death of skin cells triggered by activated Met (Fig. 4E). To examine whether Met-induced cell death occurred via cell apoptosis, the expression of an apoptosis downstream effector of caspase 3 was analyzed by Western blot. Results showed a significant reduction in pro-caspase 3 signals and a sharp increase in activated caspase 3 signals in Met-treated killer lines (Fig. 4F). Histological examination demonstrated strong apoptotic skin death in Met-treated killer line embryos. Compared to the untreated groups (Figs. 4G and 4I), the integrity of the NTR-hKikGR-expressing EVL greatly reduced after exposure of the killer line embryos to short term 10 mM Met incubation (Fig. 4H). To detect the apoptotic event *in situ*, killer line embryos aged at 24 hpf were treated with Met and TUNEL assay was performed on embryos at 48 hpf. Results showed that TUNEL^+^ cells in Met-treated killer line embryos (3548±341 mm^−2^, n = 18, Fig. 4J) have a much higher cell densities than in untreated killer line embryos (1098±668 mm^−2^, n = 19, p<0.001, Fig. 4I). This result supported the theory that apoptosis mediates loss of NTR-hKikGR^+^ signal in Met-treated killer line and this can be used as a real marker to report skin apoptosis *in vivo*.

**Figure 5:**
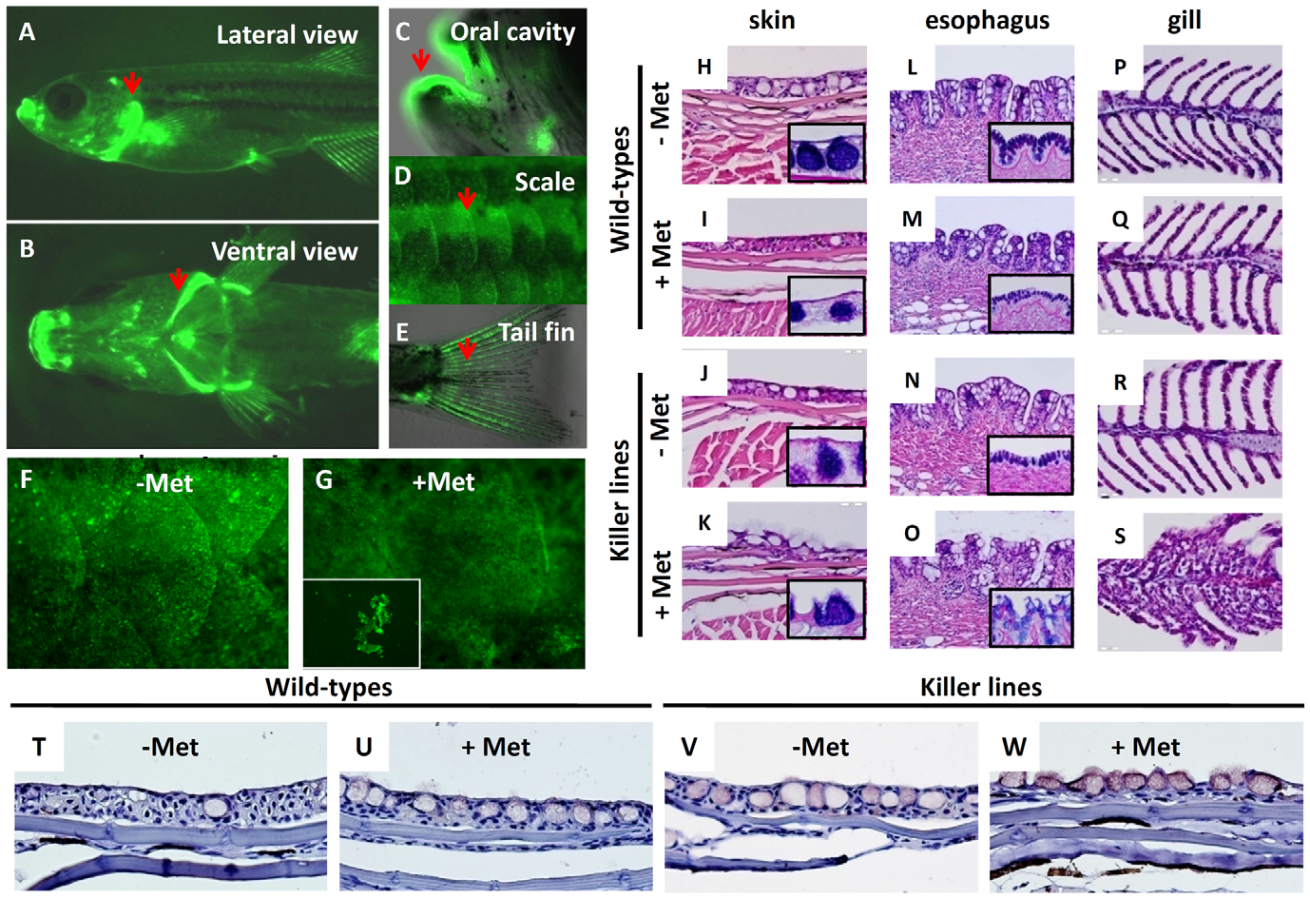
Evaluation of skin ablation by NTR/Met ablation system in adult killer line. The lateral (A) and ventral (B) views of fluorescent appearance of NTR-hKikGR fusion protein in the killer line at adult stage. Some regions like gill operculum (A, B), oral cavity (C), scale (D) and tail fin (E), which are rich in epithelial structures, showed robust fluorescent signals (heighted by arrows). (F–G) Test of the possibility of performing skin ablation in the adult killer line. Treatment of the killer line with 2.5 mM Met for three consecutive days resulted in greatly compromised skin integrity and some detached skin debris (NTR-hKikGR^+^) in the fish tank. (H–S) Histological assessment of skin integrity in wild-types or killer lines treated with or without 2.5 mM Met. Paraffin sections stained with hematoxylin and eosin showing the serial morphological changes in the regions of superficial skin (H–K), esophagus (L–O) and gill epithelium (P–S). Included for comparison, the normal epidermal histology in superficial skin (H), esophagus (L) and gill (P) before performing skin ablation. To highlight the position of mucous cells, PAS staining (blue color) in paraffin sections derived from skin (H–K) and esophagus (L–O) shown in lower right corners. (T–W) Detection of cell apoptosis in the damaged skin by activated caspase 3 antibody staining on paraffin sections (brown signals). The killer line adults were first incubated with 2.5 mM Met solution for three consecutive days to execute cell ablation and then paraffin sections, at 5 µm intervals, were cut for histological assay or immunohistochemistry. Met, metrodinazole; PAS, Periodic Acid Schiff.

In addition to embryos, skin ablation in adult zebrafish was also investigated. At adult stage, the killer line continued to express strong NTR-hKikGR^+^ signals specifically in the skin tissues. Some regions like the gill operculum (Figs. 5A and 5B), oral cavity (Fig. 5C), scales (Fig. 5D) and tail fin fold (Fig. 5E), which are rich in epithelial folding, displayed robust NTR-hKikGR^+^ fluorescent signals (indicated by arrows). Compared to embryos, the adult killer line displayed higher efficacy of NTR/Met-ablation system. Administration of Met concentration higher than 2.5 mM was too stressful and, therefore, lethal. Compared to the untreated group (Fig. 5F), some NTR-hKikGR^+^ skin cells detached from the living fish (Fig. 5G) in the killer line after continuous exposure to 2.5 mM Met for three consecutive days. The fish also presented behavioral and physiological evidences of hypoxic exposure, such as difficulty in maintaining upright posture and vertical swimming balance, bleeding, and reduced swimming mobility (Movie S1). To examine the integrity of skin before and after Met-incubation, paraffin sectioning was performed on adult killer lines treated without or with Met. Consistent with findings in embryos, the superficial skin (Fig. 5K), esophagus (Fig. 5O) and gill epithelium (Fig. 5S) of Met-treated killer lines presented significant injuries. In skin and esophagus, the disruption of superficial skin led to the release of cellular content stored in the underlying mucous cells. PAS staining characterized this material. In gill epithelium, formation of pseudobranchia-like structures, characterized by fusion between secondary lamellae, provided evidence of disruption to the epilthelium (Fig. 5S). In contrast, wild-types treated without (Figs. 5H, 5L, 5P) or with (Figs. 5I, 5M, 5Q) Met, or untreated killer lines (Figs. 5J, 5N, 5R) displayed no evidence of skin injuries. Activated caspase 3 immunostaining revealed robust apoptotic activity in the Met-treated killer line (Fig. 5W). In contrast, Met-untreated (Fig. 5T), treated wild-types (Fig. 5U), and Met-untreated killer lines (Fig. 5V) demonstrated minimal activated caspase 3 activity. These results clearly demonstrated that disruption to skin integrity in Met-treated killer line is mediated by apoptotic cell death, and also highlighted the specificity and low leakage of the NTR/Met-skin ablation system when applied in living zebrafish.

### NTR/Met-Mediated Skin Apoptosis Associates with *tp53* Activity and Oxidative Stress

**Figure 6:**
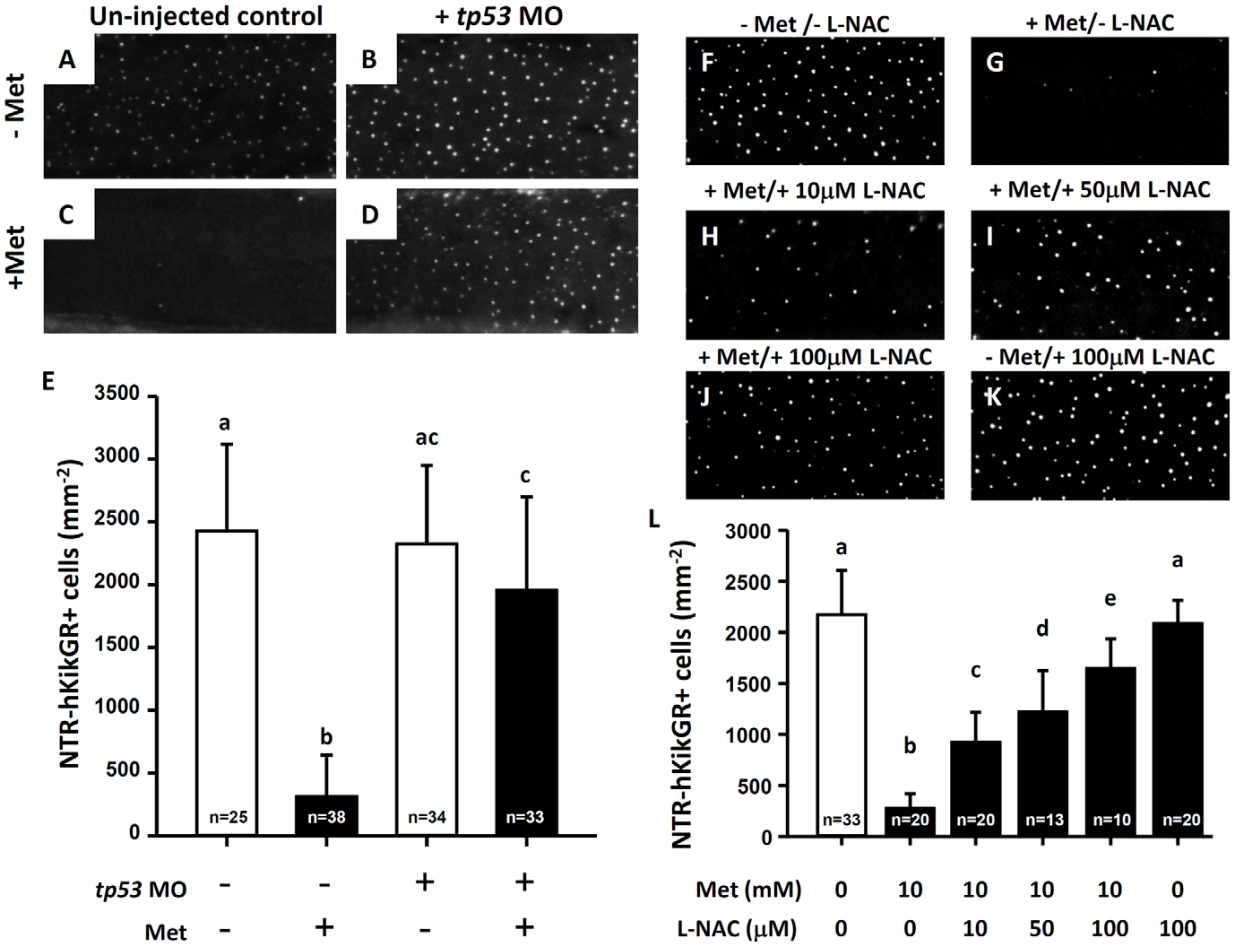
The loss of NTR-hKikGR^**+**^ fluorescent signals in Met-treated killer line correlates with *tp53* activity and oxidative stress in skin. (A–D) Test of *tp53*-dependency on the loss of NTR-hKikGR^+^ fluorescent signals by *tp53* morpholino injection. (E) Statistical comparison of the relative number of NTR-hKikGR^+^ fluorescent signals in *tp53* morphants treated with or without Met. (F–K) Test of oxidative status-dependency on the loss of NTR-hKikGR^+^ fluorescent signals by incubation with various concentrations of L-NAC as an anti-oxidant. (L) Statistical comparison of the relative number of NTR-hKikGR^+^ fluorescent signals in killer line embryos treated with Met and/or L-NAC. The cell number is presented as the mean±S.D. Different letters above the error bars indicate significant differences, as tested by one-way ANOVA with Tukey's pair-wise comparison method. L-NAC, N-acetyl-L-cysteine.

To investigate if NTR/Met-mediated skin apoptosis associates with *tp53* activity and oxidative stress protein expression level of *tp53* was knocked down by morpholino injection. Embryos were then incubated with 10 mM Met from 24 hpf onwards to trigger skin ablation. Compared to the Met-untreated killer line embryos (2426±693 mm^−2^, Figs. 6A, 6E) or *tp53*-MO injected killer line embryos (2324±627 mm^−2^, Figs. 6B, 6E), the loss of NTR-hKikGR^+^ cells in Met-treated killer line embryos (312±332 mm^−2^, Figs. 6C, 6E) was greatly attenuated by the blocking of *tp53* expression (1956±743 mm^−2^, Figs. 6D, 6E). The impact of oxidative stress on skin cell death in Met-treated embryos was then assessed. Compared to Met-treated killer line embryos (226±186 mm^−2^, Fig. 6G, 6L), the loss of NTR-hKikGR^+^ cells significantly attenuated in a dose-dependent manner after incubation with the anti-oxidative agent N-acetyl-L-cysteine (L-NAC) from 10 to 100 µM to scavenge the oxidative stress (Figs. 6H–6K). These results indicated that elevated oxidative stress mediates the loss of NTR-hKikGR^+^ signals and skin cell death in Met-treated killer line embryos in a *tp53*-dependent manner.

### The Killing Effect of NTR/Met System Specifically Targets the Superficial Skin Layer

**Figure 7:**
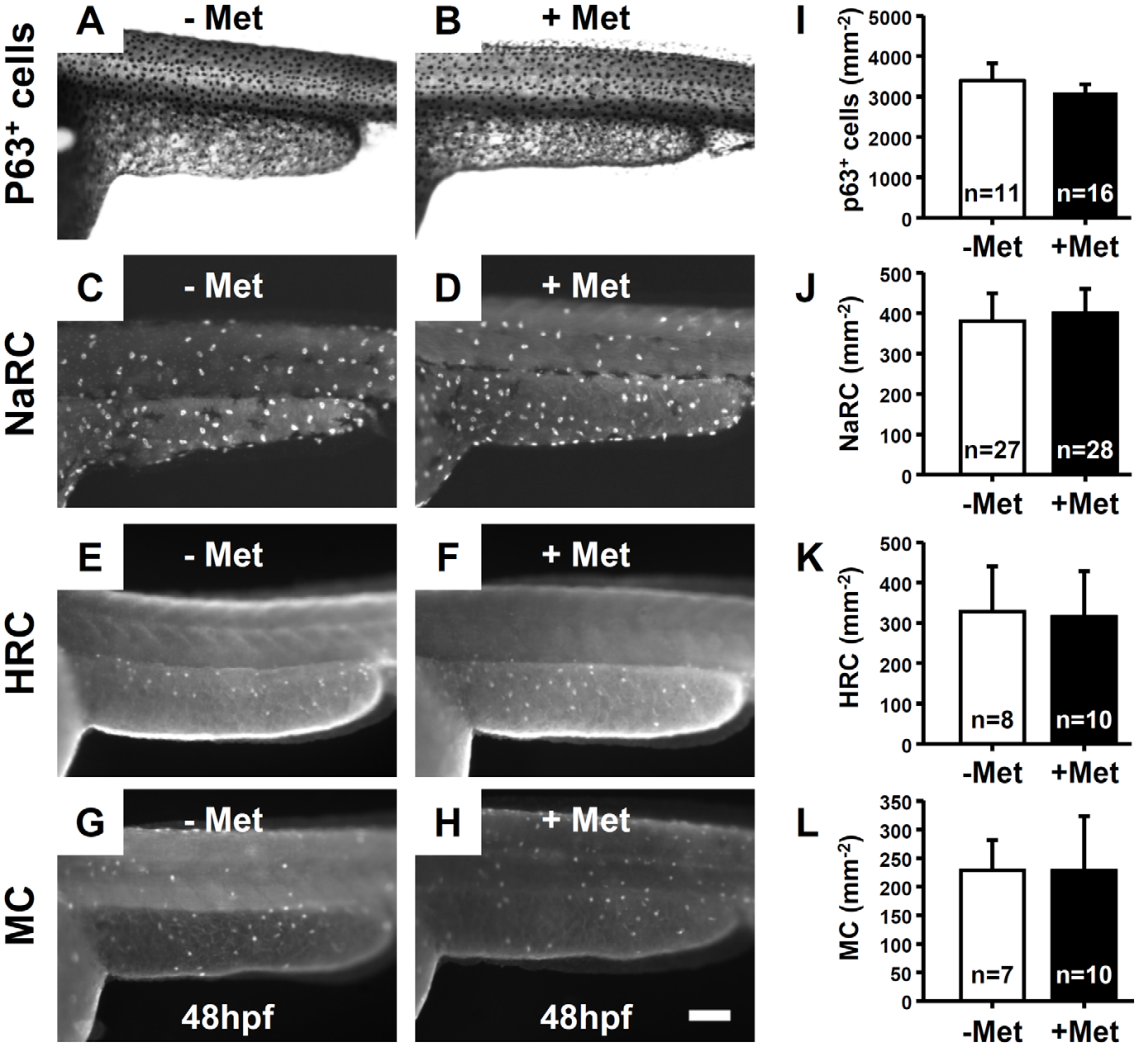
Skin ablation in Met-treated killer line specifically targeted the EVL and showed no bystander effect on the neighboring cells. Whole-mount immunostaining on killer line embryos aged at 48 hpf showing that the number of epidermal stem basal cells (A and B, stained with p63 antibody), NaRC (C and D, stained with Na,K-ATPase antibody), HRC (E and F, stained with H-ATPase antibody) and MC (G and H, stained with anterior gradient 2 antibody) did not significantly change between untreated (−Met) and treated (+Met) groups. (I–L) Statistical comparisons, using Student's *t*-test, of the relative cell numbers of p63^+^ cells, NaRC, HRC and MC between untreated (−Met) and Met-treated (+Met) groups. The cell number is presented as the mean±S.D. NaRC, Na-K-ATPase rich cell; HRC, H-ATPase rich cells; MC, mucous cells; Met, metrodinazole.

The bystander effect is a key component in tumor eradication using gene-directed enzyme prodrug therapy. Reportedly, NTR metabolizes another prototype NTR prodrug, CB1954 (5-aziridinyl-2, 4-dinitrobenzamide), to potent alkylating agents which can diffuse and kill non-NTR expressing neighboring cells by a bystander effect [48], [49]. In order to specifically ablate the superficial skin layer, the possible occurrence of the bystander effect was examined. Firstly, killer line embryos at 24 hpf were incubated with 10 mM Met and then fixed at 48 hpf to examine the integrity of other skin derived cells, such as basal epidermal cells/epidermal stem cells (Figs. 7A and 7B), Na,K-ATPase rich cells (which regulate ion homeostasis in zebrafish embryo skin, Figs. 7C and 7D), H-ATPase rich cells (which regulate acid-base and ion homeostasis in zebrafish embryo skin, Figs. 7E and 7F) and mucous cells (which secrete mucous in zebrafish embryo skin, Figs. 7G and 7H) by whole-mount antibody staining with respective antibodies as described in the Materials and Methods. The cell density for each cell type on the trunk region between untreated and Met-treated killer line embryos was quantified. The cell densities for p63^+^ cells (3402±421 mm^−2^ VS 3104±204 mm^−2^, P = 0.068, Fig. 7I), Na,K-ATPase rich cells (380±69 mm^−2^ VS 401±59 mm^−2^, P = 0.247, Fig. 7J), H-ATPase rich cells (328±112 mm^−2^ VS 316±113 mm^−2^, P = 0.822, Fig. 7K) or mucous cells (229±53 mm^−2^ VS 228±96 mm^−2^, P = 0.989, Fig. 7L) displayed no significant differences between killer line embryos treated without or with Met. These results clearly indicated that apoptosis specifically occurs in NTR-hKikGR-expressing cells and the possible bystander killing effect of NTR/Met-mediated skin ablation can be ignored.

### Testing Potential Apoptosis Modulators in Living Zebrafish Skin

**Figure 8:**
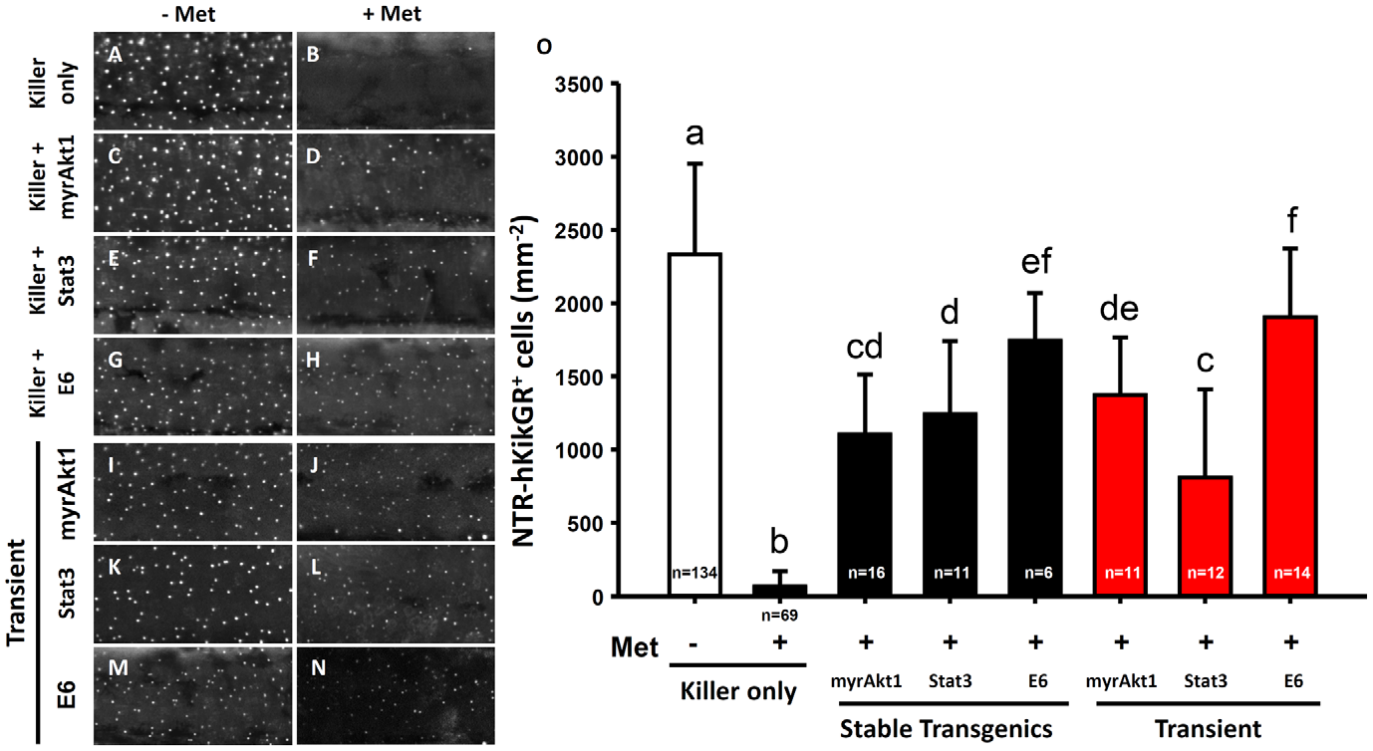
Functional validation of potential apoptosis modulators in the living killer line embryos by crossing transgenic lines or transient plasmid DNA injection. (A–H) The NTR-hKikGR^+^ fluorescent signals in embryos derived from crossing of killer line with testing line carrying human constitutively active Akt1 (myrAkt1) (C, D), mouse constitutively active Stat3 (Stat3) (E, F), or HPV16 E6 (E6) transgenes (G, H). (I–N) NTR-hKikGR^+^ fluorescent signals in killer line embryos after injection with plasmids carrying myrAkt1 (I, J), Stat3 (K, L), or E6 transgenes (M, N). (O) Statistical comparison of the relative number of NTR-hKikGR^+^ fluorescent signals in embryos derived from stable line assay (black bars) or transient assay (red bars). The cell number is presented as the mean±S.D. Different letters above the error bars indicate significant differences as tested using one-way ANOVA with Tukey's pair-wise comparison method. Met, metrodinazole.

The successful generation of a killer line which triggers apoptosis in the superficial skin layer provided the opportunity to test whether it is possible to validate potential apoptosis modulators in living zebrafish skin. Using rapid plasmid construction by Gateway recombination and germ line transmission by Tol2-mediated transgenic technology, three testing lines were created which overexpressed human constitutively active Akt1 (myrAkt1), mouse constitutively active Stat3 (Stat3), or HPV16 E6 (E6) in a superficial skin-specific manner. Previous studies documented the attenuation of UVB-induced apoptosis in skin culture cells or genetic modified mice by the three testing genes [50], [51], [52], [53]. Initially, the stable transmission and expression of the three testing transgenes in their corresponding testing lines were confirmed using PCR genotyping (Fig. S1A) and RT-PCR (Fig. S1B). Western blot analysis was performed on myrAkt1 transgenics (Fig. S1C) and real-time RT-PCR was performed on Stat3 and E6 transgenics (Figs. S1D–E) to validate the functionality of the exogenous transgenes. Detection of the activation of downstream targets confirmed that all three transgenes were functional. In myrAkt1 transgenics, the phosphorylation levels of downstream targets of GSK3α/β and p70S6K significantly elevated (Figs. S1C). The homozygotic killer line was then crossed with hemizygotic testing lines to generate double transgenics, which could be unambiguously identified based on their green skin and green heart appearance. Double transgenic embryos aged at 24 hpf were incubated with 10 mM Met to induce skin ablation and the number of NTR-hKikGR^+^ skin cells after ablation were calculated and statistically compared at 48 hpf by one-way ANOVA. In the untreated killer line (Fig. 8A) or untreated double lines derived from the crossing of the killer line and testing lines (Figs. 8C, 8E, 8G), the density of NTR-hKikGR^+^ cells maintained a consistent level of 2561±475 mm^−2^ (n = 134, Fig. 8O). Upon exposure to prodrug Met, the NTR-hKikGR^+^ cells in Met-treated killer line sharply declined to only 1% of the untreated control (32±55 mm^−2^, n = 69, Fig. 8B). However, in myrAkt1 (1104±409 mm^−2^, n = 16, Fig. 8D), Stat3 (1242±500 mm^−2^, n = 11, Fig. 8F) or E6 (1744±325 mm^−2^, n = 6, Fig. 8H) overexpression, the loss of NTR-hKikGR^+^ cells greatly attenuated when challenged with the NTR/Met ablation system.

The possibility of developing a high-throughput assay based on transient testing at F0 generation was another area investigated in the study. The plasmid DNAs carrying myrAkt1, Stat3 or E6 genes, which are driven by the *krt4* promoter, were injected into homozygotic killer lines embryos and then skin ablation was triggered by following the protocol used in stable transgenics. Consistent with results obtained from the stable lines, the transient overexpression of myrAkt1 (Fig. 8J), Stat3 (Fig. 8L) or E6 (Fig. 8N) genes in skin significantly attenuated apoptotic cell death in Met-treated killer line embryos (statistical comparisons are presented in Fig. 8O).

This study used a killer/testing binary system to quantitatively evaluate the apoptosis-modulating genes in living vertebrate skin for the first time. Researchers can use this approach in stable and transient transgenic backgrounds, providing the potential for screening of apoptosis-modulating genes in high-throughput assays in vertebrates.

## Discussion

This study established a transgenic zebrafish line which expresses the NTR-hKikGR suicide gene under the control of a superficial skin-specific promoter. Its findings demonstrated that the NTR/Met cell ablation system can be utilized to perform conditional zebrafish skin ablation in a real-time manner, and also provides a valuable tool to visualize and quantify apoptotic events *in vivo*. Successful monitoring of the fluorescent appearance of NTR-hKikGR validated the possibility of screening potential anti-apoptotic genes in living fish skin.

The NTR gene has successful clinically applications in gene-directed enzyme prodrug therapy on cancer cells [54], [55], [56] and tissue/lineage-specific cell ablation in living animals [32], [34], [35]. After administration of Met prodrug, the NTR enzyme converts the prodrug into cytotoxic alkylating agents and induces apoptosis following the formation of intrastrand DNA crosslinks on target cells. In recent years, improvements to the NTR/Met ablation system enhanced its efficacy and specificity on cell ablation. It now displays superior killing effects and specificity compared to other cell ablation systems, such as diphtheria toxin A, Kid/Kis, HSV thymidine kinase/ganciclovir, tamoxifen-inducible c-Myc, and toxic viral protein M2(H37A) [57]. To improve NTR cell sensitization, previous studies tested several amino acid substitution [30], [58], [59], codon-optimized [29] and organelle-targeted [60], [61] versions of NTR. In the killer line, incubation with Met caused ablation of at least 99% of the superficial skin cells within a short duration; from 24 to 36 hpf (Fig. 8). In the adult killer line, as low as 2.5 mM Met incubation induced massive skin ablation (Fig. 5). This lower, optimal working Met concentration is about 1/2 to 1/4 lower than previous works performed on zebrafish testis [41], ovary [42], heart, liver, or pancreas [32]. The possible reasons for the superior killing effect of the NTR/Met ablation system in this study may include that: (1) skin is the outermost tissue and directly exposed to the external chemical stimulation, therefore it is more accessible to cytotoxic damage after administration of prodrugs to the killer line; (2) the triple mutated NTR (T41Q/N71S/F124T) is the most active NTR mutation tested *in vitro* and displays a 100-fold improved specificity constant than the wild-type enzyme [30]; (3) the unique sub-cellular localization of NTR-hKikGR fusion protein might facilitate the attack of chromosomal DNA by activated Met and trigger apoptotic cell death. The appropriate subcellular localization of NTR has important consequences for suicide gene therapy [61].

Few previous studies have addressed if NTR/Met-mediated apoptosis is related to *tp53*, oxidative status or other gene activities. In an early study, Cui et al. [62] crossed BLG-NTR transgenic mice to a *tp53*-deficient mouse strain and discovered that a *tp53*-independent apoptotic pathway mediates NTR/CB1954-mediated cell ablation in mammary glands. However, in the zebrafish system in the present study, a *tp53*- and oxidative status-dependent apoptosis pathway mediated NTR/Met-mediated skin ablation in embryos. Knockdown of functional *tp53* gene expression by morpholino injection greatly attenuated skin ablation. Relief of oxidative stress by anti-oxidant reagent administration had a similar effect. Whether this difference relates to tissue- or species-differences warrants further investigation.

Based on TUNEL staining, Pai and Chen [28] discovered that UV-irradiation greatly attenuates skin cell apoptosis in prothymosin alpha transgenic embryos. However, the TUNEL assay does not detect live apoptotic events as it must be applied on fixed samples. Due to the transparency of zebrafish embryos, the UV irradiation, which targets the superficial skin layer, cannot be fully controlled. Therefore, overall skin apoptotic events might be overestimated. The routine TUNEL assay is a semi-quantitative method therefore anti-apoptotic potential among different testing genes cannot be statistically compared. Therefore, to improve on this assay, a high throughput, high specificity, *in vivo* apoptotic quantification method is necessary. This study overcame the problems of the previously mentioned study by creating a fluorescence-tagged killer line and several testing lines to validate potential anti-apoptotic gene function in living zebrafish. Due to its quantitative nature, the method could compare the anti-apoptotic potential of different testing lines. The anti-apoptotic potential of the genes validated in the study's binary system of killer/testing lines are largely consistent with previous *in vitro* and *in vivo* findings, which showed that the overexpression of human myrAkt1, mouse Stat3C, or HPV16 E6 in the skin attenuates apoptotic stress [50], [51], [52]. HPV16 E6 displayed superior anti-apoptotic effects to two other well-known survival factors, myrAkt1 and Stat3, when expressed in zebrafish skin. Previous research on cell line assays characterized HPV16 E6 as a powerful oncoprotein that can degrade p53 [63], [64]. p53 plays a key role in promotion of apoptotic cell death, therefore, down-regulation of p53 by morpholino injection or HPV16 E6 overexpression effectively attenuates skin apoptosis in zebrafish.

In the past few decades, use of genetically modified mice enabled the discovery of apoptosis modulating genes which functionally protect the skin from UV or chemical stresses. However, disadvantages of this method include low throughput, high cost and extensive manpower requirements. These factors greatly slowed the progress of investigation into apoptosis modulators. The zebrafish system has the advantages of short generation time, external fertilization, matured gateway recombination, and high efficiency Tol2-based transgenic techniques [65], [66]. Future generation of hundreds of testing lines will, therefore, be possible, along with large scale *in vivo* anti-apoptotic screening, by crossing these testing lines with the killer line. This study provided evidence to demonstrate that screening throughput can be greatly improved by performing transient assay at the F0 generation. Success in using exogenous testing genes from mammals or virus to attenuate apoptosis in zebrafish skin indicates that, although fish skin differs from its mammalian counterpart in terms of structure and cell kinetics, the underlying mechanism to control skin homeostasis is evolutionary conserved among vertebrates.

## Materials and Methods

### Plasmid Construction

Tol2 kit [65] was used to rapidly assemble expression vectors by two-fragment gateway recombination cloning. For 5′ entry cloning, 2.2 kb *krt4* promoter was amplified from genomic DNA of wild-type zebrafish by PCR with forward primer (5′-GGGGACAACTTTGTATAGAAAAGTTGCCTTCCCTTCTACTTTTGACGTCC -3′) and reverse primer (5′-GGGGACTGCTTTTTTGTACAAACTTGCCGGATCCTGTGTCTTTGAGTTGC-3′). For p5E entry cloning, the attB4 and attB1r sites were added for forward and reverse primers, respectively, at the 5′end of primers and highlighted by underlines. The PCR products were then cloned into pDONRP4-P1R (Invitrogen) by BP reaction to obtain p5E-krt4. The resulting p5E-krt4 vector contained 2.2 kb upstream regulatory sequences of *krt4* gene sufficient to target transgenes specifically expressed in skin cells [45]. For pME middle entry cloning, chimera PCR was used to fuse the mutated NTR (T41Q/N71S/F124T) in frame with hKikGR photo-convertible fluorescent protein at its C-terminus. This green-to-red photoconversion (green: 507/517; red: 583/593 (Excitation/Emission)) is sensitive to UV light (350–410 nm). Initially, the mutated NTR open reading frame (without stop codon) was amplified from plasmid pCG.Ad2v45 (kindly provided by Dr. Peter Searle) using forward primer (5′-GGGGACAAGTTTGTACAAAAAAGCAGGCTATGGATATCATTTCTGTCGC-3′) and reverse primer (5′-CGCTGGTGATCACGCTCACCATCACTTCGGTTAAGGTGATGTTT-3′). The hKikGR open reading frame was amplified from plasmid phKikGR1-MN1 [67] using forward primer (5′-AAACATCACCTTAACCGAAGTGATGGTGAGCGTGATCACCAGCG-3′) and reverse primer (5′-GGGGACCACTTTGTACAAGAAAGCTGGGTTTACTTGGCCAGCCTGGGCAGGC-3′). The two primary PCR products were mixed and subjected to a secondary round of PCR with forward primer (5′-GGGGACAAGTTTGTACAAAAAAGCAGGCTATGGATATCATTTCTGTCGC-3′) and reverse primer (5′-GGGGACCACTTTGTACAAGAAAGCTGGGTTTACTTGGCCAGCCTGGGCAGGC-3′). The *att*B1 and *att*B2 sites were added for forward and reverse primers, respectively, at the 5′end of primers and highlighted by underlines. The final PCR products were cloned into pDONR221 (Invitrogen) to generate pME-NTR-hKikGR. Finally, p5E-krt4, pME-NTR-hKikGR or pME-nlsEGFP [65] were assembled together with pTolDestR4R2pA [66] by LR reaction to create two expression vectors of pTolDest-krt4-NTR-hKikGR-pA and pTolDest-krt4-nlsEGFP-pA.

### Microinjection and Identification of Transgenic Zebrafish

Transposase RNA was synthesized *in vitro* using pCS-transposase plasmid (kindly provided by Dr. Koichi Kawakami) as a template. DNA was linearized with *Not*I at 37°C overnight and cleaned up using DNA Clean/Extraction Kit (GeneMark Inc., Taiwan). Capped mRNA was synthesized using mMESSAGE mMachine SP6 Kit (Ambion). For generation of transgenic zebrafish, expression constructs of pTolDest-krt4-NTR-hKikGR-pA (50 ng/µL) or pTolDest-krt4-nlsEGFP-pA (50 ng/µL) were mixed with *in vitro* transcribed transposases mRNA (50 ng/µL), and approximately 1–3 nL DNA solution was injected into the animal pole of one-cell stage embryos. The injected embryos were raised to adulthood and the putative founders were screened according to the green fluorescent signals in the skin of their F1 progenies. The detail procedures to generate Tg(*krt4:Hsa.myrAkt1*)^cy18^ (testing line to express human constitutively active Akt1), Tg(*krt4:Mmu.Stat3*)^cy6^ (testing line to express mouse constitutively active Stat3), Tg(*krt4:Hpv.E6*)^cy38^ (testing line to express HPV16 E6) will be available upon request. For assay of gene function at F0 generation, 5 nL of a solution containing expression constructs of either pTolDest-krt4-myrAkt1-pA, pTolDest-krt4-stat3-pA, or pTolDest-krt4-HPV16 E6-pA at 50 ng/µL concentration, together with transposases mRNA at 50 ng/µL concentration, was injected into killer line embryos at one-cell stage. The transgenic fish line nomenclatures were approved by the Zebrafish Nomenclature Committee of ZFIN (http://zfin.org). All experiments were approved by the animal use committee at Chung Yuan Christian University (approval ID: 9612).

### Applications of NTR/Met-Based Cell Ablation

Metronidazole (Sigma, M1547) was dissolved in double distilled water as a 20 mM stock and stored at −20°C in the dark. For skin ablation/recovery at embryonic stage, at least twenty wild-type or transgenic zebrafish embryos aged at 24 hpf were incubated with 10 mM Met solution (diluted with fish water) at 28.5°C. At indicated times Met solution was removed and replaced with Met-free fish water for recovery. For adult skin ablation/recovery, wild-type and transgenic fish were incubated with 2.5 mM Met solution for 3 days at 27°C, then Met was withdrawn and replaced with Met-free fish water for recovery. At each time point, at least five fish were deeply anesthetized using 0.03% MS222 and sacrificed for histological assessment.

### Histology and Immunohistochemistry

For plastic section, embryo or adult zebrafish were fixed in 4% paraformaldehyde/PBS for 1 day, washed with PBST and then dehydrated with 100% methanol for 1 day. After completely dehydration, samples were infiltrated and embedded in Technovit 7100 resin (Heraeus Kulzer). Samples were sectioned at 1–2 µm intervals and stained with hematoxylin and eosin staining kit (Merck). For paraffin section, adult zebrafish were fixed in 4% paraformaldehyde/PBS for 1 day and transferred to Davidson's solution (30% Ethyl alcohol (95%), 10% Acetic acid, 20% Formalin, and 30% double distill water) for 3 days at room temperature. This procedure decalcifies the hard tissues and also keeps the skin in good morphology. The decalcified samples were then dehydrated in ascending ethanol, cleared with Neo-clear (Merck) and embedded in Paraplast Plus/Paraplast HM (Leica) in a 7∶3 ratio (vol/vol). The paraffin embedded tissues were sectioned at 5 µm intervals with a rotational microtome (HM360, Microm) and then stained with H&E or PAS staining kit according to the manufacturer's instructions (Merck). For immunohistochemistry, the deparaffined slides were treated for antigen retrieval by incubating with 1 mM EDTA (pH 8.0) solution [68] at 95°C for 30 min. The slides were then subjected to antibody staining with primary antibodies as follows: mouse anti-human p63 (1∶200, sc-8431, Santa Cruz), rabbit anti-activated caspase 3 (1∶200, 559565, BD Biosciences), mouse anti-CK5/6 (1∶100, M7237, DAKO), rabbit anti-GFP (1∶200, sc-8334, Santa Cruz). The HRP-conjugated goat anti-mouse/rabbit IgG secondary antibodies were applied at 1∶300 dilution and the immunoreactive signals were developed using DAB as substrate (SK-4100, Vector Laboratories). Finally, the slides were counterstained with hematoxylin for 5 seconds and mounted using Neo-Mount (Merck).

### BrdU Incorporation

Adult transgenic zebrafish were systematically incubated in 10 mM BrdU (B5002, Sigma)/5% DMSO solution for 24 hr and then transferred to fresh fish water for 1 hr to remove the unincorporated BrdU. After performing fixation, decalcification and paraffin section processes according to the protocol mentioned previously, the de-paraffined slides were depurinated with 2.5N HCl for 30 min at room temperature and immersed in 0.1 M borate buffer for 10 min to enhance the detection sensitivity for the BrdU-positive cells in skin. The depurinated slides were then subjected to antibody staining with mouse anti-BrdU (1∶100, G3G4, DSHB) and HRP-conjugated goat anti-mouse IgG (Jackson ImmunoResearch).

### Whole-Mount Immunostaining

Zebrafish embryos aged at 48 hpf were fixed in 4% paraformaldehyde/PBS for 12 h at 4°C. After extensive washing in PBST, embryos were transferred to 100% methanol at −20°C for 2 h and subsequently subjected to rehydration with PBST. After blocking with 3% BSA/PBST at room temperature for 60 min, embryos were incubated at 4°C overnight with 1∶200 diluted primary antibodies as follows: mouse anti-human p63 (sc-8431, Santa Cruz), mouse anti-chicken Na^+^-K^+^-ATPase (α5, DSHB), rabbit anti-Atlantic salmon anterior gradient 2 [69], and rabbit anti-dace H-ATPase [70]. After extensive washing in PBST for 10 min, embryos were incubated with Alexa Fluor 568-conjugated goat anti-mouse/rabbit IgG (Molecular probe) or HRP-conjugated goat anti-mouse IgG (Jackson ImmunoResearch) for fluorescent or color detection of immunoreactive signals, respectively.

### Western Blot

Tail fins dissected from 15 individuals of wild-type or killer lines were pooled and homogenized in protein extraction solution (250 mM sucrose, 20 mM Hepes, 1 mM EDTA, pH 7.4, 1% protease inhibitor, Sigma). The total lysates were centrifuged at 13,000 rpm for 20 min at 4°C to remove cell debris, and the supernatant was collected for further study. The protein concentration was determined using BCA protein assay kit (23225, Thermo) and detected using Synergy HT Multi-Mode Microplate Reader (BioTek Instruments Inc., Vermont, USA). Total cellular proteins of 35 µg were separated by 12.5% SDS-PAGE and transferred to polyvinylidene difluoride (PVDF) membranes (Millipore). Following incubation with blocking solution, PVDF membranes were incubated with primary antibodies overnight and then HRP-conjugated secondary antibodies (1∶1000 dilution) for 1 hr at room temperature. The primary antibodies and their dilution factor were as follows: mouse anti-hKikGR (1∶1000, M129-3, MBL), rabbit anti-activated caspase 3 (1∶1000, 559565, BD Biosciences), mouse anti-pan cytokeration (1∶1000, C2931, Sigma), rabbit anti-human phosphorylated GSK3α/β (pY279/pY216) (1∶1000, 2309-1, Epitomics), rabbit anti-human phosphorylated stat3 (pY705) (1∶5000, 2236-1, Epitomics), rabbit anti-human phosphorylated p70 S6 kinase (pT421/pS424) (1∶5000, 1135-1, Epitomics), and mouse anti-β actin (1∶2000, sc-69879, Santa Cruz). After a series of washing, locations of protein were revealed by incubating in the WEST-ZOL PLUS solution (iNtRON Biotechnology, Korea) for 2 min at room temperature in the dark room, and the images were acquired using FUJIFILM LAS 3000 Imaging Analyzer (FUJIFILM, Taiwan).

### TUNEL assay

For TUNEL assay, embryos aged at 18 hpf were pre-treated with PTU and then processed to PFA fixation at 48 hpf. Following washing in PBST for 10 min they were then stored in 100% methanol at −20°C for over 2 h. Embryos were incubated with 3% H_2_O_2_/MeOH for 10 min at room temperature. Subsequently, embryos were rinsed two times with PBS and incubated with labeling solution, 10 µL of enzyme solution plus 90 µL of label solution at 37°C for 2 h following the kit instructions (Roche Applied Sciences). Embryos were then washed three times in PBS, for 5 min each time, at room temperature. Subsequently, embryos were incubated with 100 µL of converter POD at 37°C for 30 min. Embryos were rinsed three times in PBS and incubated with DAB solution (SK-4100, Vector Laboratories) for 5 min.

### RT-PCR

Thirty embryos aged at 3 dpf from wild-type or testing lines were collected and homogenized in RNAzol RT (RN190, MRC, Inc) with Bullet Blender (Next Advance, Inc) tissue lyser to isolate total RNA according to the manufacturer's instructions. Total RNA concentration was determined by spectrophotometry, and the RNA quality was checked by running electrophoresis in RNA-denatured gels. For RT-PCR, 1 µg of total RNA was reverse-transcribed with RevertAid first cDNA synthesis kit (K1622, Fermentas) and then PCR was performed with SYBR green dye according to the manufacturer's instructions. The primer sequences used to perform RT-PCR and the PCR amplicon size are listed in Table S1.

### *tp53* Morpholino Oligo Injection

To achieve the maximal knock-down effect, *tp53* MO (5′-AGAATTGATTTTGCCGACCTCCTCT-3′, Gene Tools), at a concentration of 5 ng/embryo, was injected into yolks at the one-cell stage. The specificity and efficacy of *tp53* MO has been validated previously [71].

### Image Acquisition, Skin Cell Quantification and Statistics

Representative DAB-stained or fluorescent images were acquired using an upright microscope (BX51, Olympus) equipped with a digital camera (DP72, Olympus) or a dissecting microscope (SMZ1500, Nikon) equipped with a cool CCD (Evolution VF). For quantifying the relative density of skin cells or TUNEL^+^ cells, the original images were processed using Photoshop CS3 software to select a region of interest (ROI) at 300 µm×150 µm dimensions. The total cell number in this ROI was calculated using ImageJ software (http://rsbweb.nih.gov/ij/) and statistically compared using t-test or one-way ANOVA.

## Supporting Information

Figure S1 **Validation of the transmission, expression and function of testing lines in stable transgenics using genotyping, RT-PCR and Western blot analysis.** Assay of the stable transmission and expression of transgenes in different testing lines at the DNA and mRNA levels by genotyping (A) and RT-PCR (B), respectively. The relative position of the primers used to perform genotyping or RT-PCR is illustrated by red arrows. The predicted amplicon size is also illustrated in the right panel. The plasmid architectures were highlighted in the upper panel. (C) Western blot assay on protein lysates extracted from adult tail fins showing highly phosphorylated Akt downstream targets of GSK3α/β and 70S6K in myrAkt1 testing line. β-actin served as a protein loading control. (D) Quantitative real-time RT-PCR showed that overexpression of pro-survival genes of Stat3 down-regulates the pro-apoptotic genes *bida* and *puma* in transgenic embryos aged at 48 hpf. (E) Quantitative real-time RT-PCR showed that overexpression of virus oncogene of HPV16 E6 up-regulates the cell cycle-related genes *ccne* and *cdk2* in transgenic embryos aged at 48 hpf.
(PDF)

Table S1 **The PCR amplicon size and primer sequences used to perform RT-PCR.**
(XLSX)

Movie S1 **Swimming behavior assessment of killer line treating without (−Met, left tank) or with (+Met, right tank). Met, metrodinazole.**
(WMV)
