# Supplementary figures and images for: Establishment of a Transgenic Zebrafish Line for Superficial Skin Ablation and Functional Validation of Apoptosis Modulators In Vivo
Source: PLoS One. 2011 May 31;6(5):e20654. doi: 10.1371/journal.pone.0020654 (PMC3105106; doi:10.1371/journal.pone.0020654)

# [Fig. S1 ]

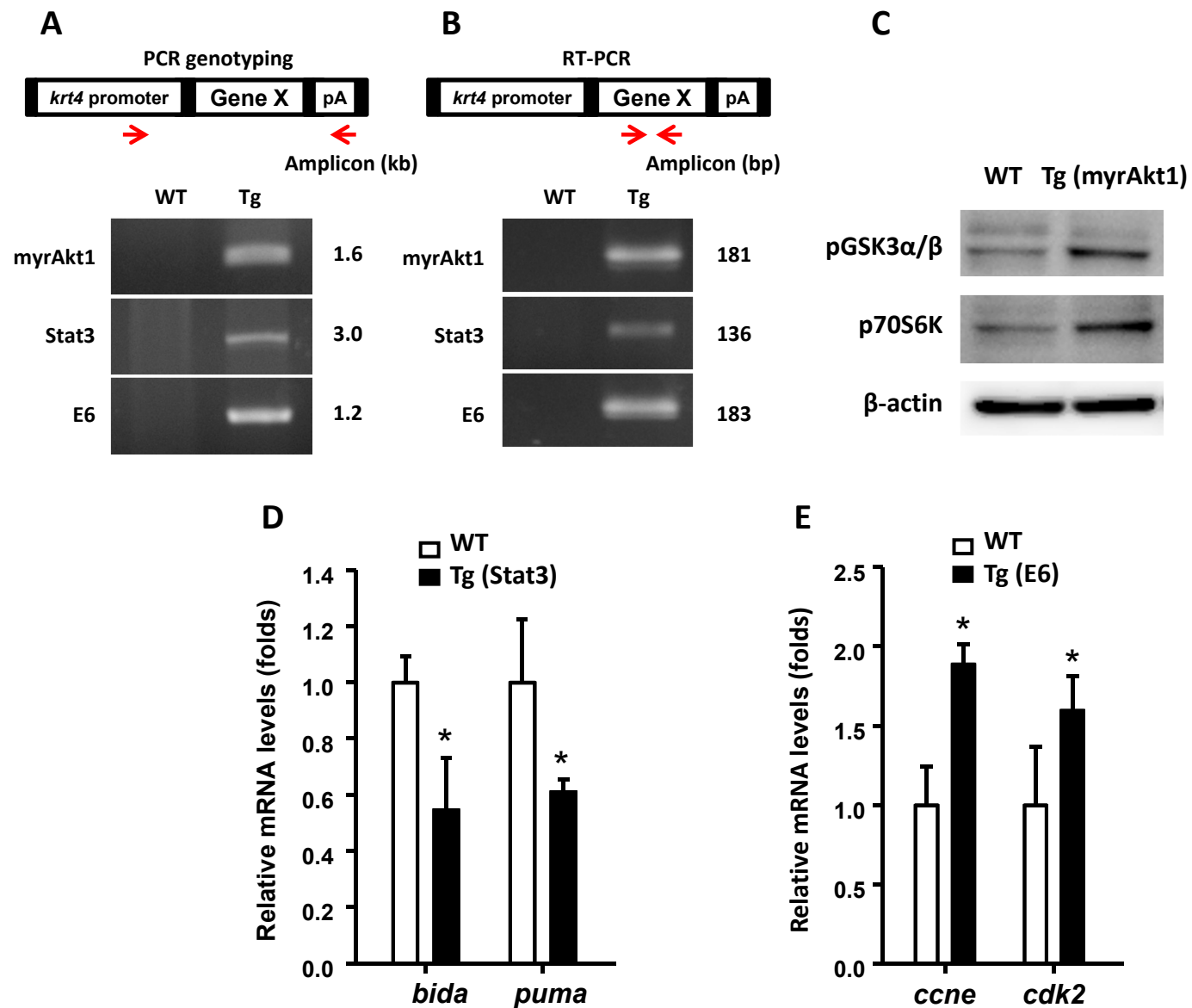

Supplement: Figure S1 — Validation of the transmission, expression and function of testing lines in stable transgenics using genotyping, RT-PCR and Western blot analysis. Assay of the stable transmission and expression of transgenes in different testing lines at the DNA and mRNA levels by genotyping (A) and RT-PCR (B), respectively. The relative position of the primers used to perform genotyping or RT-PCR is illustrated by red arrows. The predicted amplicon size is also illustrated in the right panel. The plasmid architectures were highlighted in the upper panel. (C) Western blot assay on protein lysates extracted from adult tail fins showing highly phosphorylated Akt downstream targets of GSK3α/β and 70S6K in myrAkt1 testing line. β-actin served as a protein loading control. (D) Quantitative real-time RT-PCR showed that overexpression of pro-survival genes of Stat3 down-regulates the pro-apoptotic genes bida and puma in transgenic embryos aged at 48 hpf. (E) Quantitative real-time RT-PCR showed that overexpression of virus oncogene of HPV16 E6 up-regulates the cell cycle-related genes ccne and cdk2 in transgenic embryos aged at 48 hpf. (PDF) [file pone.0020654.s001.pdf]
